# Deep statistical modelling of nanopore sequencing translocation times reveals latent non-B DNA structures

**DOI:** 10.1093/bioinformatics/btad220

**Published:** 2023-06-30

**Authors:** Marjan Hosseini, Aaron Palmer, William Manka, Patrick G S Grady, Venkata Patchigolla, Jinbo Bi, Rachel J O’Neill, Zhiyi Chi, Derek Aguiar

**Affiliations:** Department of Computer Science and Engineering, University of Connecticut, Storrs, CT 06269-4155, United States; Department of Computer Science and Engineering, University of Connecticut, Storrs, CT 06269-4155, United States; Department of Computer Science and Engineering, University of Connecticut, Storrs, CT 06269-4155, United States; Institute for Systems Genomics and Department of Molecular and Cell Biology, University of Connecticut, Storrs, CT 06269-3003, United States; Department of Computer Science and Engineering, University of Connecticut, Storrs, CT 06269-4155, United States; Department of Computer Science and Engineering, University of Connecticut, Storrs, CT 06269-4155, United States; Institute for Systems Genomics and Department of Molecular and Cell Biology, University of Connecticut, Storrs, CT 06269-3003, United States; Department of Statistics, University of Connecticut, Storrs, CT 06269-4120, United States; Department of Computer Science and Engineering, University of Connecticut, Storrs, CT 06269-4155, United States

## Abstract

**Motivation:**

Non-canonical (or non-B) DNA are genomic regions whose three-dimensional conformation deviates from the canonical double helix. Non-B DNA play an important role in basic cellular processes and are associated with genomic instability, gene regulation, and oncogenesis. Experimental methods are low-throughput and can detect only a limited set of non-B DNA structures, while computational methods rely on non-B DNA base motifs, which are necessary but not sufficient indicators of non-B structures. Oxford Nanopore sequencing is an efficient and low-cost platform, but it is currently *unknown* whether nanopore reads can be used for identifying non-B structures.

**Results:**

We build the first computational pipeline to predict non-B DNA structures from nanopore sequencing. We formalize non-B detection as a novelty detection problem and develop the GoFAE-DND, an autoencoder that uses goodness-of-fit (GoF) tests as a regularizer. A discriminative loss encourages non-B DNA to be poorly reconstructed and optimizing Gaussian GoF tests allows for the computation of *P*-values that indicate non-B structures. Based on whole genome nanopore sequencing of NA12878, we show that there exist significant differences between the timing of DNA translocation for non-B DNA bases compared with B-DNA. We demonstrate the efficacy of our approach through comparisons with novelty detection methods using experimental data and data synthesized from a new translocation time simulator. Experimental validations suggest that reliable detection of non-B DNA from nanopore sequencing is achievable.

**Availability and implementation:**

Source code is available at https://github.com/bayesomicslab/ONT-nonb-GoFAE-DND.

## 1 Introduction

**Figure 1.**
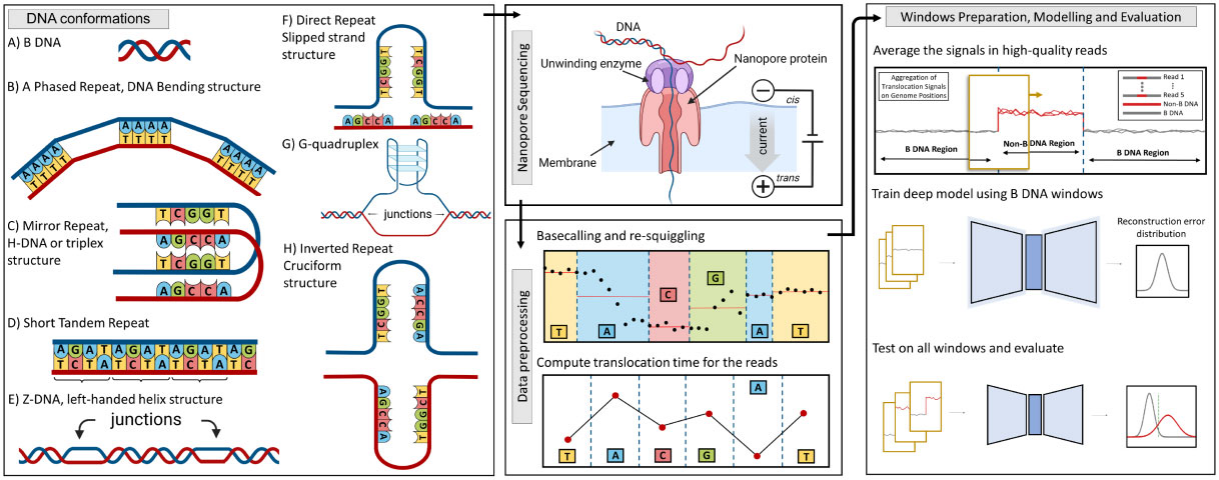
Non-B DNA prediction workflow. Left: Non-B DNA motifs and conformations: (A) B-DNA, (B) A-phased repeat, (C) mirror repeat, (D) short tandem repeat, (E) Z-DNA, (F) direct repeat, (G) G-quadruplex, (H) inverted repeat. Middle: Nanopore sequencing is followed by basecalling, re-squiggling, and computation of TTs. Right: TT windows are extracted, followed by modelling and evaluation.

The three-dimensional conformation of DNA is most commonly associated with a right-handed double helix structure called B-DNA (Fig. 1A) (Watson and Crick 1953). However, alternative, non-canonical conformations may form in the presence of specific DNA nucleotide base motifs (Cer et al. 2011). The DNA hexamer CGCGCG, which may form a left-handed double helix termed z-DNA (Rich and Zhang 2003), was among the first such non-canonical DNA conformations discovered (Wang et al. 1979). Additional non-canonical structures such as cruciforms formed by inverted repeats (Lilley 1980), G-quadruplexes (G4s) formed in guanine-rich sequences (Sen and Gilbert 1988), and others (Drew and Travers 1985; Koo et al. 1986; Mirkin and Frank-Kamenetskii 1994; Sinden et al. 2007) were subsequently discovered and collectively termed ‘non-B’ DNA (Fig. 1B–H) (Jovin 1976).

Observations of non-B DNA conservation across species (Yadav et al. 2008) and characterizations of their molecular functions (Wells et al. 1977, 1988) suggest that non-B DNA plays an important role in cellular processes. Due to their presence in promoters, origins of replication, and telomeres, non-B DNA structures are believed to play critical roles in gene regulation and telomere stability (Boyer et al. 2013). Recent work has shown that non-B DNA may play a vital role in the segregation of genetic material, e.g. the establishment of centromeric chromatin (Kasinathan and Henikoff 2018; Talbert and Henikoff 2022). Furthermore, non-B DNA is associated with increased mutability (Georgakopoulos-Soares et al. 2018), DNA repair inhibition, genomic instability (Zhao et al. 2010; Wang and Vasquez 2014), and oncogenesis (Ray et al. 2013), which supports non-B DNA as an emerging anti-cancer therapeutic target (Kosiol et al. 2021).

Early efforts to catalogue non-B DNA conformations characterized nucleotide base motifs that provide compatible environments for non-B DNA formation. There are more than 10 known non-B DNA conformations (Svozil et al. 2008), 7 of which have well characterized DNA base motifs (Cer et al. 2012b). While these motifs cover approximately 13% of the genome (Guiblet et al. 2021), they are ‘necessary’ but ‘not sufficient’ indicators of non-B DNA conformation. A small number of genomic regions identified by non-B DNA sequence motifs are occupied by non-B DNA structures at any given time; e.g. less than 10% of Z-DNA, G4, and H-DNA regions identified by DNA base motifs were found to form non-B DNA structures in activated mouse B cells (Kouzine et al. 2017). Additionally, non-B DNA structures are transient since their formation and stability depend on the conditions within the cell (Zhao et al. 2010; Guiblet et al. 2021).

Current experimental techniques for identifying non-B DNA are low-throughput, expensive, and limited to identifying a subset of non-B DNA structures. Computational approaches offer a low-cost alternative, but rely on non-B DNA motif labels, which are noisy indicators of non-B conformation (Kouzine et al. 2017). Oxford Nanopore Technology (ONT) sequencing is a portable, efficient, and low cost platform for sequencing DNA and calling methylation (Petersen et al. 2019), but it is currently *unknown* whether ONT reads can be used for identifying non-B structures (Guiblet et al. 2018).

### 1.1 Our contributions

In this work, we develop the first computational pipeline and a novel deep statistical model for predicting non-B DNA structures from ONT sequencing. Specifically, we model the differential time it takes for B and non-B DNA to pass through the nanopore [i.e. translocation times (TTs); Fig. 1, middle]. Since ground truth non-B DNA structures are unavailable and B-DNA far outnumbers non-B DNA (Guiblet et al. 2021), we formalize non-B detection as a novelty detection problem in a hypothesis testing framework. We develop the GoFAE-DND, an autoencoder (AE) that uses goodness-of-fit (GoF) test statistics as a regularizer such that encoded B-DNA is indistinguishable from Gaussian (Fig. 1, right). A discriminative loss encourages B-DNA to be reconstructed well while non-B DNA is reconstructed poorly; the optimization of GoF tests ensures that the empirical distribution of encoded B-DNA is regular. We use the B-DNA empirical distributions of reconstruction error and Mahalanobis distance (MD) to compute *P*-values for samples with non-B DNA motifs, which are interpreted as B or non-B DNA after controlling for false discovery rate (FDR). Based on whole genome ONT sequencing of NA12878, we show that there exist significant deviations in the TTs of non-B DNA motifs compared with B-DNA. We construct the first TT simulator and demonstrate the efficacy of GoFAE-DND through comparisons with novelty detection methods using both experimental and synthetic data. Experimental validation shows that GoFAE-DND achieves improved detection accuracy in simulations, calls more non-B DNA novelties at a controlled FDR, and produces stable G4 novelties. In total, our work suggests that the reliable detection of non-B DNA from ONT sequencing is achievable.

## 2 Preliminaries

Nanopore sequencing from ONT is a portable, relatively low cost, and efficient technology that can sequence native DNA and RNA molecules of arbitrary read length (Deamer et al. 2016; Gamaarachchi et al. 2022). The technology relies on a biological nano-scale protein pore (nanopore) that serves as an electrically resistant biosensor. A helicase protein unzips double-stranded DNA and then a motor protein translocates the nucleic acid through the pore in a step-wise manner (typically at a rate of ∼450 bases per second). The presence of the DNA molecule causes an obstruction in the pore, and consequently a deflection in the current. These changes in the current, or ‘squiggles’, are used to assign nucleotide base labels to the typically 5- or 6-mers that are present in the pore (Bao et al. 2021; Wang et al. 2021). Results are provided in FAST5 format, which contains the raw electrical signal data and supporting metadata.

### 2.1 Non-B DNA structures

The non-B database contains DNA motif annotations for seven non-B structures (Cer et al. 2012b). ‘DNA bending’ non-B structures cause the DNA molecule to bend at an angle and form in A-phased repeat motifs—four or six consecutive A-T base pairs without a TpA step (Fig. 1B) (Stefl et al. 2004). ‘H-DNA’ structures form from mirror repeats (Fig. 1C) (Vikash et al. 2013). Short tandem repeat motifs consist of 2–7 nt that repeat and contribute to forming slipped strands and other non-B DNA conformations (Fig. 1D) (Mirkin and Mirkin 2007; Butler 2011). ‘Z-DNA’ has a left-handed double helical structure and a zigzagging sugar–phosphate backbone. The bases in Z-DNA alternate between an ‘anti’ and ‘syn’ orientation about *N*-glycosidic bonds such as (CG_**·**_CG)${}_{n}$ and (CA_**·**_TG)${}_{n}$ (Fig. 1E). ‘Slipped strand’ or ‘hairpin’ structures form in regions with direct repeats and microsatellites (Fig. 1F). ‘G-quadruplexes’ (G4s) have a high guanine composition and a helical shape containing square planar structures called guanine tetrads (Fig. 1G) (Bacolla and Wells 2004). Finally, ‘cruciforms’ are similar to slipped strands, but their inverted repeat motifs cause each strand to fold at the repeat centre of symmetry and reconstitute as an intra-molecular B-helix capped by a single-stranded loop (Fig. 1H) (Bacolla and Wells 2004).

### 2.2 Determining non-B structures experimentally

Efficient detection of non-B structures *in vivo* has been a longstanding challenge in experimental biology. Early experimental techniques for detecting non-B structures used gel electrophoresis to identify Z-DNA (Kladde et al. 1994). More recent approaches used spectroscopic assays (Georgakopoulos-Soares et al. 2022) or engineered antibodies with subsequent deep sequencing for mapping G4 DNA structures (Lam et al. 2013). Permanganate footprinting combined with high-throughput sequencing has been used to identify single-stranded DNA in Z-DNA, G4, stress-induced duplex destabilized DNA, and H-DNA structures (Kouzine et al. 2017). The G4 ChIP-seq assay combines G4 targeted chromatin immunoprecipitation and high-throughput sequencing for genome-wide G4 structure prediction (Hänsel-Hertsch et al. 2016, 2018). While these techniques yield direct evidence of a subset of non-B DNA structures, several considerations limit their widespread adoption. First, these techniques tend to be low-throughput, expensive, and may not be representative of an *in vivo* cellular environment, which is problematic since non-B structural conformation is dependent on cellular conditions that can vary among samples (e.g. supercoiling, transcription, and ionic concentrations) (Guiblet et al. 2021). Second, experimental assays are specific to a subset of non-B DNA types and typically require both knowledge of the prospective site and *a priori* assumptions of the type of non-B DNA to be assessed (Kladde et al. 1994; Lam et al. 2013; H**ä**nsel-Hertsch et al. 2016, 2018; Georgakopoulos-Soares et al. 2022).

### 2.3 Computational prediction of non-B structures

Early computational methods for identifying non-B DNA relied primarily on DNA sequence motifs capable of forming structures (Huppert and Balasubramanian 2005, 2007; Cer et al. 2012a). Subsequent score-based and probabilistic methods targeted the detection of G-quadruplexes (Bedrat et al. 2016; Hon et al. 2017). The most recent methods use deep neural networks to predict non-B DNA from one-hot encoded DNA sequences (Rocher et al. 2021). These methods are based primarily on DNA sequence motifs, which are necessary but insufficient for formation and are not available for all non-B DNA structures (Cer et al. 2012b). Furthermore, the precise coordinates of the non-B DNA structures are difficult to determine from sequence alone and are transient based on their structural stability (Zhao et al. 2010; Guiblet et al. 2021).

A promising new approach emerged based on the observation that the polymerization speed in PacBio Single-Molecule Real-Time (SMRT) technology is affected by DNA methylation (Flusberg et al. 2010). The SMRT device emits a fluorescent pulse when a nucleotide is detected in a sequence; the time interval between two such pulses is known as the interpulse duration (IPD) (Eid et al. 2009). Guiblet et al. (2018) showed that there exists a significant divergence between the IPDs in non-B DNA motif regions compared with B-DNA regions and suggested the open problem of computing non-B structures from ONT sequencing.

Given the dramatic increase in genome-scale data produced using ONT platforms, and in particular ultra-long sequencing data that support telomere-to-telomere level genome assembly, we sought to develop a parallel strategy for identifying non-B DNA structure by their effects on sequencing speeds (TTs) in ONT devices (Nurk et al. 2022). Unlike SMRT sequencing whose sequencing speed is determined by polymerization, ONT devices record a measurement of current at a predefined sampling rate and then aggregate the measurements into *strides*, which are the smallest length of measurement accepted by the basecaller and represent a single base translocation. Recent work on detecting DNA methylation from the charge in ONT reads provides evidence that DNA modifications can affect ONT TTs (Stoiber et al. 2017; Liu et al. 2019; McIntyre et al. 2019; Ni et al. 2019).

## 3 Non-B DNA prediction: problem formulations

Prior work on the computational prediction of non-B DNA structure used motif labels from the non-B database in a classification setting. However, by assuming that DNA structures absent of a non-B motif form B-DNA, then approximately ∼87% of the genome can be labelled as B-DNA and the non-B DNA structure prediction problem can be cast as an anomaly detection (or more precisely, novelty detection) problem. ‘Anomaly detection’ refers to identifying observations that do not conform to the expected behaviour characterized by the majority of the data (Chandola et al. 2009). These aberrations may be due to a number of factors like measurement noise or emerging new behaviours; hence the modelling methodology is problem dependent. ‘Outlier detection’ is a specific anomaly detection task that is appropriate when anomalies are rare and located in regions of low density. In contrast, ‘novelty detection’ methods generally do not require anomalies to be rare nor in regions of low density. The novelty detection perspective yields several advantages over supervised approaches: (i) the non-B database labels are noisy—only a small fraction of regions annotated as non-B DNA based on sequence motifs are occupied by non-B DNA structures at any given time (Cer et al. 2012b; Kouzine et al. 2017); (ii) even if high-quality labeling for non-B DNA were available, substantially more B-DNA samples are available; and (iii) unknown non-B structures or non-B DNA without sequence motifs cannot be modelled by a supervised approach.

We consider DNA segments identified as containing a non-B DNA motif as a mixture from two populations: (i) segments without non-B DNA structure, which we assume to be distributed the same as B-DNA, and (ii) segments with a non-B DNA structure, which we assume to have a different distribution from B-DNA. Formally, let *x*, *y*, and *s* be random variables where *x* is a sequence of non-negative TTs, y∈{0,1} indicates the absence or presence of structure (y=1 if there is structure, y=0 otherwise), and s∈{0,1} indicates the absence or presence of a motif. Denote the joint distribution of the triplet (x,y,s) as p(x,y,s). Let $X^{B}={\left\{x_{i}\right\}}_{i=1}^{n_{B}}$ denote a set of $n_{B}$ realizations of B-DNA coming from p(x|y=0,s=0) and $X^{N}={\left\{x_{i}\right\}}_{i=1}^{n_{N}}$ denote the set of $n_{N}$ realizations of non-B DNA coming from p(x|s=1). Then the ‘ONT non-B structure prediction problem’ is to distinguish between y=1 and y=0 in $X^{N}$ given the full data $X=X^{B}\cup X^{N}$ and labels *s*.

## 4 Deep statistical modelling of non-B DNA

We begin by describing our processing pipeline for computing TTs. Then, we describe GoFAE-DND, a new novelty detection method based on large-scale multiple hypothesis testing and discriminative goodness-of-fit autoencoders (GoFAEs) (Palmer et al. 2022).

### 4.1 Nanopore read processing pipeline

**Figure 2.**
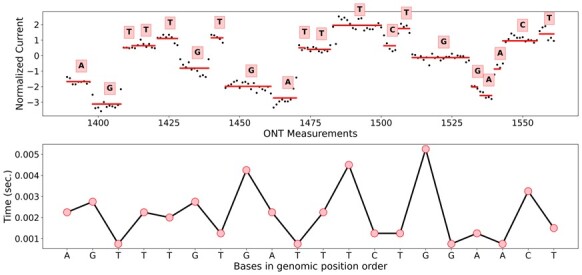
TT computation using Tombo. The re-squiggled current measurements (top) are converted to TTs (bottom) based on the number of samples assigned to each nucleotide.

Sequence bases are called from the raw ONT current using Albacore, which generates an event table that describes the DNA context in the nanopore (Loman et al. 2015). Subsequently, we re-squiggle the FAST5 output of Albacore using Tombo, a statistical method that detects base modifications in nanopore current signal (Stoiber et al. 2017). Briefly, the re-squiggling algorithm segments the raw current signal into events and calls nucleotide bases using the current and a reference genome for correcting spurious variation (Fig. 2, top). The Tombo segmentation provides current measurements at the base-level, unlike Albacore, which assumes the block stride attribute remains fixed, which enables the computation of TTs (Fig. 2, bottom). For each position on the Tombo-mapped reads, we compute the time duration in seconds as the ratio of the number of current measurements to the ONT sampling rate.

### 4.2 GoFAE-DND: the GoFAE for discriminative novelty detection

Our approach first learns a representation of the B-DNA sequences and then conducts large scale multiple hypothesis testing on sequences with non-B DNA motifs. Each sequence $x_{i}$ is assessed under the null hypothesis $H_{0}:x_{i}∼p(x|y=0,s=0)$, i.e. whether it comes from the same distribution as B-DNA. The null distribution is derived from B-DNA regions and deviations from it should indicate non-B DNA structures. However, we emphasize that this is a statistical assessment and ‘not’ proof that a non-B DNA structure is present. Such a conclusion can only be verified by a direct observation of the structure. Nevertheless, our approach can prioritize putative non-B regions for subsequent biological experimentation.

Since we frame the ONT non-B structure prediction problem as novelty detection, a model for the non-novel (B-DNA) data is needed. We posit two axiomatic requirements for a model F(x) to support hypothesis testing of high dimensional TT data.

1.
F(x) should retain information about *x* otherwise a hypothesis test on F(x) is not informative of the population distribution of X. Formally, we impose the condition ∃G, such that G(F(x))≈x.
2. The distribution of F(x) should be regular for *x* from p(x|y=0,s=0), in the sense that it has properties similar to a normal distribution, e.g. being unimodal and relatively smooth, or having no concentration on any manifold of a lower dimension. Hypothesis testing typically involves regular null distributions, the archetypal example being the normal distribution.

In practice, *F* and *G* will be neural networks parameterized by θ and ϕ, respectively. The GoFAE provides an ideal architecture that satisfies requirements (1) and (2) (Palmer et al. 2022). Given a minibatch of size *m* of training data, ${\left\{x_{i}\right\}}_{i=1}^{m}\subset X^{B}$, GoFAE optimizes the los*s* for parameters θ and ϕ, regularization parameter λ, a test statistic *T*, and reconstruction loss function d(⋅,⋅). The sign of λ is determined by the test statistic. GoFAE uses GoF hypothesis tests as a regularizer at the minibatch level and selects λ based on a test on the uniformity of the local GoF *P*-values (Donoho et al. 2004).


(1) $$\frac{1}{m}\sum_{i=1}^{m}d(x_{i},G_{\phi }(F_{\theta }(x_{i})))\pm \lambda T(\{F_{\theta }{\left(x_{i}\right)}_{i=1}^{m}),$$

**Figure 3.**
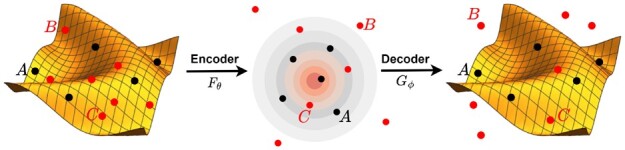
Illustration of GoFAE-DND. Left: The starting point of B-DNA (•) and non-B DNA (•) distributed on the data manifold. Middle: The GoF regularizer shapes the B-DNA data in the latent space to be indistinguishable from Gaussian. Right: The encoded samples are mapped back to the data space where the discriminative loss encourages B-DNA to reconstruct well (close to the manifold, e.g. point A) and non-B DNA poorly (pushed away from the manifold). Points B and C depict samples labelled with a non-B DNA motif with an unknown structure. Since there is no constraint on how non-B DNA data is encoded, it can overlap with the Gaussian-like B-DNA (point C) or encode to the tails (point B), which are pressured to reconstruct away from the data manifold. While the discriminative loss tries to push non-B DNA labelled points far away, points like C that are similar enough to B-DNA should remain close throughout the mapping. If this were not the case, the model would reconstruct B-DNA poorly.

Instead of only using B-DNA, we modify the reconstruction loss in Equation (1) for our novelty detection formalization by leveraging non-B DNA. Discriminative autoencoders use data from two classes and learn a manifold which reconstructs positive samples well while encouraging negative samples to be pushed away from the manifold (Razakarivony and Jurie 2014). We arrive at the discriminative GoFAE for novelty detection, or GoFAE-DND (Fig. 3), which optimizes the empirical loss: where the $x_{i}$’s are samples from $X^{B}$, the $z_{i}$’s are samples from $X^{N}$, and $n_{B}$ and $n_{N}$ are the numbers of samples from $X^{B}$ and $X^{N}$, respectively. Note that, the regularization term $\lambda T(\{F_{\theta }(x_{i})\})$ is only applied to those $n_{B}$ samples where $x_{i}\in X^{B}$ (discussed further in Section 4.2.1). Pseudo-code for fitting the GoFAE-DND is given in Supplementary Algorithm S1. The architecture is located in Supplementary Table S1 with hyper-parameter and training details discussed in Supplementary Section A.3. Following the notation of Equation (2), we show that in the large sample limit, the optimal GoFAE-DND is a classifier for $X^{N}$ versus $X^{B}$ based on a likelihood ratio.


(2) $$\begin{array}{c} \frac{1}{n_{B}}\sum_{i=1}^{n_{B}}d(x_{i},G_{\phi }(F_{\theta }(x_{i})))\\ +\frac{\omega }{n_{N}}\sum_{i=1}^{n_{N}}\max(0,\delta -d(z_{i},G_{\phi }(F_{\theta }(z_{i})))\pm \lambda T(\{F_{\theta }(x_{i})\}), \end{array}$$

Proposition 1. *Given* δ>0*and let* $K(x)=\frac{1}{\delta }d(x,G_{\phi }(F_{\theta }(x)))$*and* J(K)=E[δK(x)]+ωE[max(0,δ−δK(x))]*, i.e. the expected loss of the first two terms of**Equation (2)*. *Then the optimal*$K^{⋆}=\arg\min_{K}J(K)$*is determined by the likelihood ratio* p(x,s=1)/p(x,y=0,s=0)*. Explicitly*,

(3) $$K^{⋆}(x)=\{\begin{array}{cc} 0 & \;\text{if}\;cp(x,s=1)\leq p(x,y=0,s=0),\\ 1 & \;\text{if}\;cp(x,s=1)>p(x,y=0,s=0), \end{array}$$

*where* c=ωp(y=0,s=0)/p(s=1)*. Furthermore, if* $\left(1+c)p(x,y=0,s=1)<\frac{1}{2}p(x,y=0\right)$*, andthen* $\tilde{K}(x)\geq K^{*}(x)$.


(4) $$\tilde{K}(x)=\{\begin{array}{cc} 0 & \text{if}\;2cp(x,y=1)\leq p(x,y=0),\\ 1 & \text{if}\;2cp(x,y=1)>p(x,y=0), \end{array}$$

Note that by construction, $K^{*}$ alone cannot be considered a classifier of y=0 versus y=1 because it involves *s*. In contrast, $\tilde{K}$ can be considered such a classifier in addition to a likelihood ratio test. However, since $\tilde{K}(x)\geq K^{*}(x)$, whenever $K^{*}(x)=1$, $\tilde{K}(x)=1$, so $K^{*}$ can, in fact, be interpreted as a classifier of y=0 versus y=1, whose calls are a subset of the calls made by $\tilde{K}$. Therefore, $K^{*}$ is less powerful than $\tilde{K}$ (i.e. there is a cost for using *s* to make a decision).

#### 4.2.1 Controlling for false discoveries

For each window $z\in X^{N}$, we test the null hypothesis $H_{0}$: $\left\{F_{\theta }(z),d(z,G_{\phi }(F_{\theta }(z)))\right\}$ follows the same distribution as $\left\{F_{\theta }(x),d(x,G_{\phi }(F_{\theta }(x)))\right\}$, which we denote as $\left\{z,d,F_{\theta },G_{\phi }\right\}$ for brevity. We use the empirical distribution of $\left\{x,d,F_{\theta },G_{\phi }\right\}$ as the null distribution. To do this, we first learn $F_{\theta }$ and $G_{\phi }$ on a training and validation set. A non-discriminative model would train and validate on a subset of $X^{B}$, but the GoFAE-DND also includes a subset of $X^{N}$. Training regularization is based on the Shapiro–Wilk GoF test for normality on $F_{\theta }(x)$. Since using the empirical distribution of $\left\{x,d,F_{\theta },G_{\phi }\right\}$ from the training and validation data may cause overfitting, we use the empirical distribution of $\left\{x,d,F_{\theta },G_{\phi }\right\}$ derived from the held-out test data in $X^{B}$, denoted $X_{te}^{B}$.

The last step of GoFAE-DND performs multiple hypothesis testing. We consider the $L_{1}$ reconstruction error $\left||x-G_{\phi }(F_{\theta }(x))|\right|$ and MD ${\left[{\left(F_{\theta }(x)-\mu \right)}^{\prime }{\hat{\Sigma }}^{-1}(F_{\theta }(x)-\mu )\right]}^{1/2}$ as test statistics, where μ and $\hat{\Sigma }$ are the sample mean and covariance matrix, respectively, of F(x) over $x\in X_{te}^{B}$. Since novelties within B-DNA will affect the covariance matrix estimate, we use a robust version of MD estimated from minimum covariance determinant estimators (Hubert et al. 2018), which applies best for elliptically distributed data; hence, the Gaussian regularizer. Supplementary Algorithm S2 describes how reconstruction error and MD are combined to create the statistic of interest and consequently the empirical null distribution. Supplementary Algorithm S3 dictates how the statistic is computed on new observations.

Given a test statistic *S*, for each $z\in X^{N}$, we calculate the *P*-value of $S(\{z,d,F_{\theta },G_{\phi }\})$ under the empirical distribution of $S(\{x,d,F_{\theta },G_{\phi }\})$. The corresponding lower-tail *P*-value is calculated a*s*


(5) $$p=\frac{\#\{x\in X_{te}^{B}:S(\{x,d,F_{\theta },G_{\phi }\})\leq S(\{z,d,F_{\theta },G_{\phi }\})\}}{\left|X_{te}^{B}\right|}.$$

An upper-tail *P*-value is calculated as 1−lower-tail P-value. As a general guideline, we use a lower-tail *P*-value if the empirical distribution of $S(\{z,d,F_{\theta },G_{\phi }\})$ is shifted to the left compared with $S(\{x,d,F_{\theta },G_{\phi }\})$ and use an upper-tail *P*-value if the shift is to the right. Finally, with all the *P*-values of $z\in X^{N}$ calculated, given a defined FDR control level α, we apply Benjamini–Hochberg multiple testing procedure to all the *P*-values of $S(\{z,d,F_{\theta },G_{\phi }\})$, $z\in X^{N}$ (Benjamini and Hochberg 1995).

## 5 Results

We evaluated our methods using both synthetic and experimental data. For experimental sequencing data, we consider ONT whole genome sequencing data for NA12878 (Gamaarachchi et al. 2022). These data include over 9*M* reads sequenced by PromethION using LSK109 ligation library prep and two flow cells to generate ∼30× genome coverage. The genomic positions of non-B DNA base motifs were extracted from the non-B database (Cer et al. 2012b). Since the true locations of non-B DNA structures are unknown, we also generated synthetic data using a novel TT simulator (described in detail in the following sections).

### 5.1 Preprocessing

We generated samples in the experimental data by first isolating genomic intervals (windows) that matched non-B DNA motifs from the non-B database. Based on the distribution of motif-lengths in the non-B database (Supplementary Fig. S1), we constructed 100 bp windows centred around each non-B DNA motif in the human genome (version hg38). Motifs that were longer than 100 bp had their flanking segments trimmed and motifs shorter than 100 bp were padded with their flanking B-DNA. We removed any two non-B DNA windows with a non-zero intersection. In the remaining windows, we extracted the median TTs for each position and strand from the aligned ONT reads. To account for noise in TTs, we removed windows with a coverage less than 5 reads and performed robust scaling (subtracted the median and divided by the interquartile range of the training set) to form the non-B DNA dataset $X^{N}$. B-DNA windows were calculated from genomic intervals without non-B DNA motifs to construct $X^{B}$ (100 bp windows and a median of 5 or more reads; for additional processing details, see Supplementary Sections B.1–B.5).

During training, novelty detection methods typically assume far more non-novelty samples than novelties. We split the B-DNA (non-novelty) data $X^{B}$ into training, validation, and test sets $X_{tr}^{B}$, $X_{v}^{B}$, and $X_{te}^{B}$ with the proportions of 30% (322 275 samples), 20% (214 850 samples) and 50% (537 125 samples), respectively. The non-B DNA data $X^{N}$ was split into $X_{tr}^{N}$, $X_{v}^{N}$, and $X_{te}^{N}$ such that each non-B type had 10%, 10%, and 80% of their total data in training, validation, and testing, respectively. We split the validation B-DNA into two sets: $X_{v}^{P}$ and $X_{v}^{-P}$. The poison set $X_{v}^{P}$ is added to $X_{v}^{N}$ to create a mixed validation set $X_{v}^{M}$ for evaluating false positives. The non-poison validation B-DNA ($X_{v}^{-P}$) was used to estimate the empirical null distribution. We use an equal number of non-B and B samples in $X_{v}^{M}$.

### 5.2 Evaluation criteria and model selection

The experimental data labels are noisy and prior work suggests that less than 10% of non-B DNA motif windows for Z-DNA, G4, and H-DNA form non-B DNA structures at any given time (Kouzine et al. 2017). Therefore, we consider an approach commonly employed in eQTL studies (Aguiar et al. 2018); after model training, we compute an empirical distribution of scores on $X_{te}^{B}$, which forms an empirical null distribution. We generate scores for each $x\in X_{te}^{N}$ and compute a *P*-value analogously to Equation (5). Finally, performance is evaluated based on the number of significant predictions at an FDR control level of α=0.2 using the Benjamini–Hochberg multiple testing procedure (Benjamini and Hochberg 1995). The $F_{1}$ score is used to evaluate each method in the simulated data since the true labels are known.

For model selection in both the experimental and simulated data, we define a grid over hyperparameters (Supplementary Table S2). We perform model validation over these hyperparameters at increasing α levels from 0.25 to 0.95 increasing by 0.05 until at least one configuration has at least one non-B novelty called. Let the number of novelties called in $X_{v}^{N}$ and $X_{v}^{P}$ be $\hat{\text{TP}}$ and $\hat{\text{FP}}$, respectively. We select the model that maximizes $\frac{\hat{\text{TP}}}{1+\hat{\text{FP}}}$.

### 5.3 Non-B signal in nanopore

**Figure 4.**
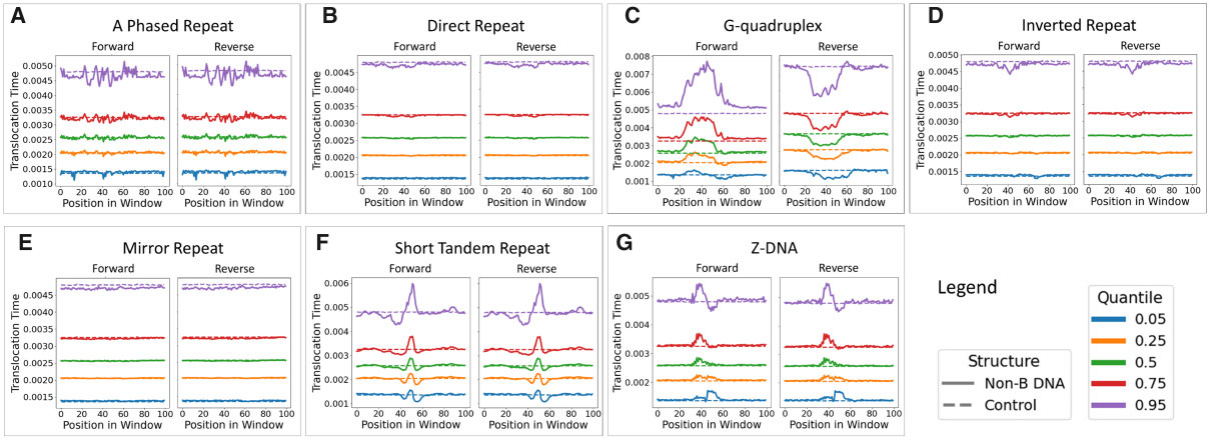
Comparison between B and non-B DNA TT windows. The {0.05,0.25,0.5,0.75,0.95} quantiles of TTs for (solid lines). (A) A phased repeats, (B) direct repeats, (C) G-quadruplexes, (D) inverted repeats, (E) mirror repeats, (F) short tandem repeats, and (G) Z-DNA windows show deviation from B-DNA control windows (dashed line). The *x*-axis gives the position relative to the window start and the *y*-axis is the TT. The number of non-B DNA motif windows can be found in the Supplementary Data (Supplementary Table S4).

We first investigated whether ONT sequencing demonstrated a signal in DNA motif regions associated with non-B DNA to justify statistical modelling. We considered the {0.05,0.25,0.5,0.75,0.95} quantiles of TTs in 100 bp windows centred around non-B DNA motifs. All non-B DNA motif windows exhibited significant deviations from B-DNA windows across all non-B types (Fig. 4). Motif regions for A phased repeats, Z-DNA, G-quadruplex, and short tandem repeats exhibited the largest deviations across all quantiles compared with the B-DNA controls. GC-content for Z-DNA and G4 was enriched compared with baseline, whereas repetitive non-B motifs were depleted (Supplementary Fig. S2 and Supplementary Table S3).

**Figure 5.**
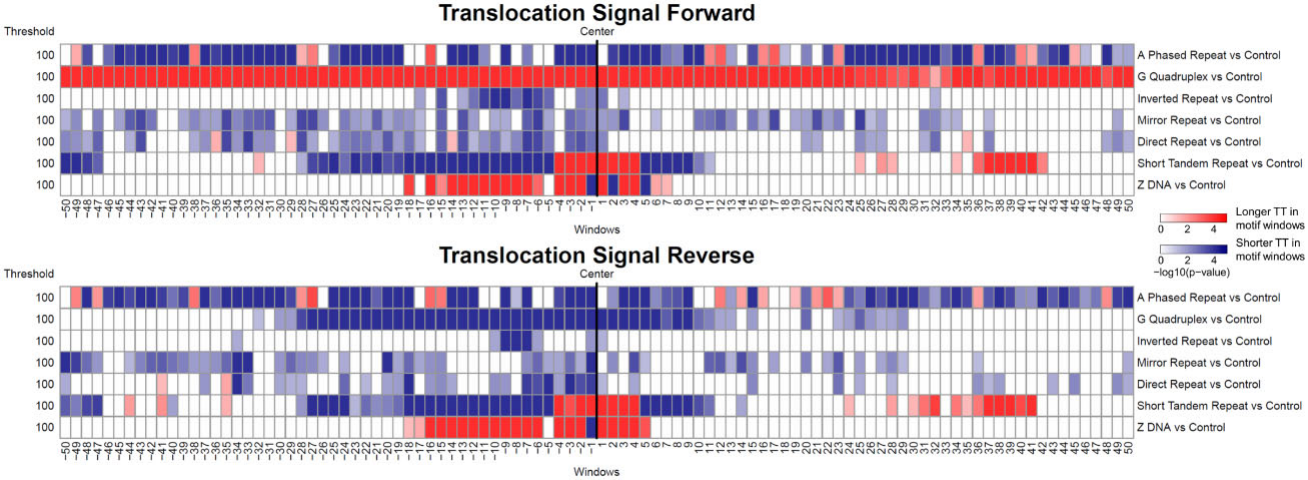
Significance of non-B TT deviation versus control. The deeper the red (blue) colour the longer (shorter) the TT in non-B DNA motif windows compared with the B-DNA control. Window positions (*x*-axis) are relative to the window centre.

Next, we evaluated whether the TTs in non-B DNA motif windows significantly diverged from B-DNA TTs. Significance was evaluated using interval wise testing (IWT) (Cremona 2018), which is a hypothesis testing procedure for time series data often applied to the functional data analysis of omics data (Guiblet et al. 2018). IWT performs a non-parametric permutation test to evaluate differences in time series measurements between two region datasets (see Supplementary Section A.5 for details); here, we used IWT to evaluate where TT curve distributions differ at nucleotide resolution between non-B and B-DNA windows. The TTs for most non-B DNA motif windows significantly diverged from B-DNA windows for both forward and reverse strands (Fig. 5). The directionality of the non-B TT deviation from B-DNA is consistent with the quantile plots (Fig. 4). These results, while largely in agreement with prior work on PacBio IPD values (Guiblet et al. 2018), have a unique signature suggesting that ONT and PacBio sequencing would be complementary for non-B DNA detection.

### 5.4 Experimental validation

We compared the performance of GoFAE-DND with isolation forests (IF), the local outlier factor (LOF) algorithm, and one-class support vector machines (SVMs) on the NA12878 ONT data (see Supplementary Section A.4 for a description of these methods). Preliminary validation studies showed that all methods benefited from the removal of flanking B-DNA, thus we trained and tested each model on the central 50 bp region of the windows. After model selection and training, we generated IF, LOF, and one class SVM scores from $X_{te}^{B}$ to serve as an empirical null distribution. Based on model outputs for $X_{te}^{N}$, we computed an empirical *P*-value for each method and non-B DNA type. Additionally, we reduced the B-DNA (‘not’ the non-B DNA) dataset size for the non-neural network methods due to prohibitively high training times (see Supplementary Section B.5).

At an FDR control level α=0.2, SVM and GoFAE-DND generated the most novelties, with GoFAE-DND yielding the most predictions for all non-B types besides G4 (Supplementary Table S5). Although there is conflicting evidence on the number of non-B structures that typically form (Kouzine et al. 2017; Tu et al. 2021), the SVM and GoFAE-DND predicted novelties for G4 and short tandem repeats are likely overestimates. Interestingly, GoFAE-DND performs uniquely well on A phased repeats and Z-DNA, for which significant deviations from B-DNA exist (Figs 4 and 5). All methods perform poorly on mirror and direct repeats, which is expected given that the TT signal is not well preserved across quantiles (Fig. 4).

We also evaluated the stability of G4 novelties using Quadron (Table 1). Quadron leverages non-B DNA motif annotations and G4-seq (Chambers et al. 2015), a profile of G-Quadruplex formation in the human genome based on Illumina sequencing mismatch scores, to produce scores reflecting G4 stability (Sahakyan et al. 2017). Quadron scores are strongly correlated with biophysical assays of stability, and exhibit bimodal separation corresponding to ‘weak’ (Quadron score ≤ 19) and ‘strong’ (Quadron score > 19) G4 stability. Besides LOF which did not produce any G4 novelties, IF identified the smallest set of novelties, but with the most weakly and strongly stable G4s (Table 1). Both SVM and GoFAE-DND produced substantially more G4 novelties than IF, but stability was notably smaller. In total, these results suggest the each method is finding biologically relevant signal for G4s.

**Table 1.** Proportion of G4 novelties that have weak or strong stability as estimated from Quadron.

	G4 calls	Weak stability (%)	Strong stability (%)
GoFAE-DND	11 334	63.4	43.8
SVM	12 364	63.2	42.6
IF	3003	67.4	48.9

### 5.5 Computational benchmarking on simulations

Due to their distinct TT signatures, we developed mathematical functions for generating TT samples of G4s and short tandem repeats that resemble the experimental data (Supplementary Figs S3 and S4). Short tandem repeat and G4 samples were generated from sinusoidal and quadratic function, respectively, with N(0,1) Gaussian noise added (see Supplementary Section B.5 for details). We generated three G4 and short tandem repeat datasets (six in total) where we adjusted the ratio of true non-B DNA to B-DNA that were labelled as non-B DNA ∈{0.05,0.1,0.25}. Each dataset contained 200 000 B-DNA windows (sampled from N(0,1)) and 20 000 non-B DNA windows. Data splits were similar to the experimental setting (see Supplementary Section B.5).

**Figure 6.**
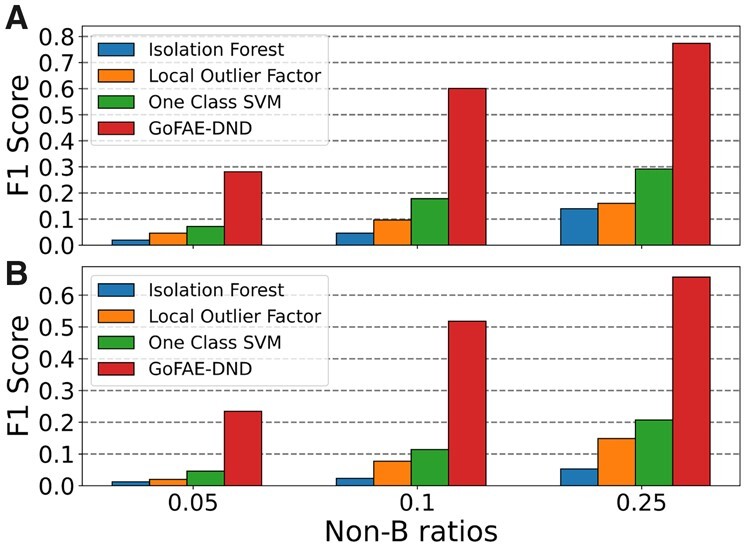
A comparison of $F_{1}$ scores for non-B novelty detection methods in synthetic data for (A) G4 and (B) short tandem repeats.

We evaluated GoFAE-DND and competing novelty detection methods on the simulated data (Fig. 6 and Supplementary Table S6). For both datasets, GoFAE-DND achieved the highest $F_{1}$ scores (0.2812 and 0.2344) for the most challenging non-B ratio (0.05). As the ratio increase, $F_{1}$ scores consistently improved, but GoFAE-DND maintained the highest $F_{1}$ by a wide margin, followed by SVM, LOF, and then IF. We also compared our method to several classifiers: support vector classifiers, logistic regression, K-nearest neighbours, Gaussian processes, and random forests (Supplementary Fig. S4 and Supplementary Table S6). Before running the classifiers, we balanced the datasets. GoFAE-DND, SVM, and LOF largely outperform the classification models, which supports our novelty detection problem formulation. Lastly, we visualized the data samples processed by GoFAE-DND with respect to reconstruction error and MD (Supplementary Fig. S5). Qualitatively, we can visually separate the false positives (which act more like outliers) from the true-positive novelties that cluster together.

## 6 Discussion

We framed non-B DNA prediction as a novelty detection problem since motif annotations are noisy and to accommodate non-B DNA types without DNA motifs. However, with prior caveats, a supervised approach may be warranted if reliable experimentally derived labels can be secured. Also, the formation of non-B DNA is associated with other cellular processes, and thus TTs can be combined with additional features like mutation rates (Georgakopoulos-Soares et al. 2018) or genomic position (Kouzine et al. 2017) to enhance computational methods.

The quantile plots demonstrated a strong non-B DNA signal compared with B-DNA controls in ONT sequencing. However, these plots mask the variability within and between non-B DNA motifs. For example, non-B DNA motifs vary in mean length from 15.3 (short tandem repeats) to 49.9 (mirror repeats), with high standard deviations, e.g. 38.4 for direct repeats, which is larger than its mean of 35.0 (Supplementary Fig. S1). This suggests that methods making assumptions about a standard motif length may not generalize to all non-B DNA samples. Our analysis of TTs also showed a large diversity in signal, which may be caused by: (i) differential effects of non-B DNA structures on TTs; (ii) variability in the conditions for forming non-B DNA or maintaining them throughout the sequencing process; and (iii) aggregate statistics might be masking substantial signals in the tails.

We aggregated TTs across reads using the median to be robust to noise. This was motivated by observing significant outliers in single bases of both B and non-B DNA. These outliers could be biological [e.g. methylation is known to affect IPDs (Flusberg et al. 2010)] or technical artefacts. For example, shorter DNA molecules (<100 bases) travel fast through the pore, sometimes going entirely undetected (Plesa et al. 2013). Modelling of ONT in the presence of this noise is a major challenge both in non-B DNA prediction and traditional computational tasks like assembly (Lu et al. 2016). Lastly, with respect to chemical constraints on non-B formation, K${}^{+}$, Na${}^{+}$, and Li${}^{+}$ are critical ingredients in nanopore sequencing (Vu et al. 2019) and cations are known stabilizers of DNA generally (Pina et al. 2022), and G4s specifically (Largy et al. 2016). Additional biochemical evidence is needed to support the retention of non-B conformations throughout the ONT sequencing process.

## 7 Conclusions

In this work, we developed the first computational pipeline to predict non-B DNA structures from ONT sequencing. We demonstrated that the TTs in ONT sequencing data yield a signature in non-B DNA motifs that is significantly divergent from B-DNA. These results were largely congruent and complementary with prior work using IPDs in PacBio data. Motivated by this signature, we formulated the prediction of genomic locations occupied by non-B structures as a novelty detection problem; to solve this problem, we developed GoFAE-DND, an autoencoder that leverages GoF tests to satisfy important regularity conditions and to support multiple hypothesis testing. Our discriminative learning and hypothesis testing framework accommodates the noisy labels that are characteristic of non-B DNA annotations. We constructed the first TT simulator for ONT and demonstrated the efficacy of GoFAE-DND through comparisons with competitive novelty detectors and classifiers using both experimental and synthetic data. While further experimentation is required to validate this workflow *in vivo*, our results suggest that modelling TTs in ONT data is an effective strategy for large-scale detection of non-B DNA.

## Supplementary Material

btad220_Supplementary_Data

## Data Availability

The data underlying this article are available in the Sequence Read Archive at https://trace.ncbi.nlm.nih.gov/Traces/?view=run_browser&acc=SRR15058166, and can be accessed with identifier 15188789 (Gamaarachchi et al. 2022). Source code for the computational pipeline and GoFAE-DND is available at https://github.com/bayesomicslab/ONT-nonb-GoFAE-DND.
