# Supplementary material for: Deep statistical modelling of nanopore sequencing translocation times reveals latent non-B DNA structures
Source: Bioinformatics. 2023 Jun 30;39(Suppl 1):i242–51. doi: 10.1093/bioinformatics/btad220 (PMC10311326; doi:10.1093/bioinformatics/btad220)
Supplement: btad220_Supplementary_Data [file btad220_supplementary_data.pdf]

Supplementary Materials

A Supplementary Methods

A.1 Proofs

Proposition 1. *Given* $\delta > 0$ *and let* $K(x) = \frac{1}{\delta} d(x, G_\phi(F_\theta(x)))$ *and* $J(K) = \mathbb{E}[\delta K(x)] + \omega \mathbb{E}[\max(0, \delta - \delta K(x))]$*, i.e. the expected loss of the first two terms of Eq. (2). Then the optimal* $K^\star = \arg\min_K J(K)$ *is determined by the likelihood ratio* $p(x, s = 1)/p(x, y = 0, s = 0)$*. Explicitly,*

$$K^\star(x) = \begin{cases} 0 & \textit{if } cp(x, s = 1) \leq p(x, y = 0, s = 0), \\ 1 & \textit{if } cp(x, s = 1) > p(x, y = 0, s = 0), \end{cases} \tag{3}$$

where $c = \omega p(y = 0, s = 0)/p(s = 1)$*. Furthermore, if* $(1 + c)p(x, y = 0, s = 1) < \frac{1}{2} p(x, y = 0)$*, and*

$$\tilde{K}(x) = \begin{cases} 0 & \textit{if } 2cp(x, y = 1) \leq p(x, y = 0), \\ 1 & \textit{if } 2cp(x, y = 1) > p(x, y = 0), \end{cases} \tag{4}$$

then $\tilde{K}(x) \geq K^*(x)$.

Proof. Since

$$\begin{aligned} J(K) &= \mathbb{E}[\delta K(x)] + \omega \mathbb{E}[\max(0, \delta - \delta K(x))] \\ &= \int \delta K(x) p(x \,|\, y = 0, s = 0)\, dx \quad (6) \\ &+ \omega \int \max(0, \delta - \delta K(x)) p(x \,|\, s = 1)\, dx, \quad (7) \end{aligned}$$

minimizing $J(K)$ is equivalent to minimizing

$$\int K(x) p(x, y = 0, s = 0)\, dx + c \int \max(0, 1 - K(x)) p(x, s = 1)\, dx. \tag{8}$$

where we defined $c = \omega p(y = 0, s = 0)/p(s = 1)$. Define the following likelihood ratio

$$h(x) = \frac{p(x, s = 1)}{p(x, y = 0, s = 0)}. \tag{9}$$

By substituting $p(x,s = 1)$ with $h(x)p(x,y = 0,s = 0)$, the second integral in (8) can be written as

$$\int \max(0, 1 - K(x)) p(x, s = 1)\, dx = \int \max(0, 1 - K(x)) h(x) p(x, y = 0, s = 0)\, dx.$$

The minimization of (8) becomes finding $K(x)$ to minimize

$$\int [K(x) + ch(x) \max(0, 1 - K(x))] p(x,y = 0,s = 0)\, dx. \tag{10}$$

Given $\beta > 0$, let $l_\beta(z) = z + \beta \max(0, 1 - z)$ for $z \geq 0$. Then,

$$l_\beta(z) = \begin{cases} z + \beta(1 - z) & \text{if } 0 \leq z \leq 1 \\ z & \text{if } z \geq 1 \end{cases}$$

When $\beta \leq 1$ then $\min_{z \geq 0} l_\beta(z) = l_\beta(0) = \beta$. When $\beta > 1$ then $\min_{z \geq 0} l_\beta(z) = l_\beta(1) = 1$. Applying this to $K(x) + ch(x) \max(0, 1 - K(x))$ with $\beta = ch(x)$, the minimizing $K(x)$ for the integral in Eq. (10) is

$$K(x) = \begin{cases} 0 & \text{if } cp(x, s = 1) \leq p(x, y = 0, s = 0) \\ 1 & \text{if } cp(x, s = 1) > p(x, y = 0, s = 0) \end{cases}$$

giving the first part of the proposition.

To prove the second part, first, note that

$$p(x,s=1) = p(x,y=1,s=1) + p(x,y=0,s=1)$$
$$= p(x,y=1) + p(x,y=0,s=1)$$

Therefore, $cp(x,s=1) > p(x,y=0,s=0)$ is equivalent to

$$p(x,y=0,s=0) - cp(x,y=0,s=1) < cp(x,y=1) \tag{11}$$

Since $p(x,y=0,s=0) = p(x,y=0) - p(x,y=0,s=1)$, (11) is equivalent to

$$p(x,y=0) - (1+c)p(x,y=0,s=1) < cp(x,y=1). \tag{12}$$

Now assume that $(1+c)p(x,y=0,s=1) < \frac{1}{2}p(x,y=0)$. Then Eq. (12) implies

$$\frac{1}{2}p(x,y=0) < cp(x,y=1) \tag{13}$$

By construction

$$\tilde{K}(x) = \begin{cases} 0 & \text{if } 2cp(x,y=1) \le p(x,y=0) \\ 1 & \text{if } 2cp(x,y=1) > p(x,y=0). \end{cases}$$

It follows that whenever $K^*(x) = 1$, (12) holds, and so (13) holds, giving $\tilde{K}(x) = 1$. Since both K^* and $\tilde{K}$ take only take either 0 or 1, then $\tilde{K}(x) \ge K^*(x)$.

A.2 Fitting the `GoFAE-DND`

Pseudo-code for fitting the `GoFAE-DND` is given in Algorithm S1. Riemannian optimization is needed for fitting part of $\theta = [\Xi, \Theta]$ and is detailed in (Palmer *et al.*, 2022).

Algorithm S1 `GoFAE-DND` Algorithm

Input: $\mathbf{D}_1 \subset \mathcal{X}^B, \mathbf{D}_2 \subset \mathcal{X}^N$, test T, $\lambda \geq 0$, $\delta > 0$, $\omega \in [0,1]$, J iter.

Initialize: Ξ, Θ, ϕ
while $j < J$ **do**
  Sample mini-batch $\mathbf{X}_1 \subset D_1$, $|\mathbf{X}_1| = m_1$
  Sample mini-batch $\mathbf{X}_2 \subset D_2$, $|\mathbf{X}_2| = m_2$
  $\mathbf{V}_1 = F_{\boldsymbol{\Xi}}(\mathbf{X}_1)$, and $\mathbf{V}_2 = F_{\boldsymbol{\Xi}}(\mathbf{X}_2)$
  $\mathbf{Y}_1 = \mathbf{V}_1\Theta$, and $\mathbf{Y}_2 = \mathbf{V}_2\Theta$
  if If T uses projection **then**
    $T^\star = T(\mathbf{Y_1 u})$, where $\mathbf{u} \in \mathcal{S}$, unit sphere ▷ Alternatively can sample an orthonormal basis and use multiple projections.
  else
    $T^\star = T(\mathbf{Y}_1)$
  $\hat{\mathbf{X}}_1 = G_{\boldsymbol{\phi}}(\mathbf{Y}_1)$, and $\hat{\mathbf{X}}_2 = G_{\boldsymbol{\phi}}(\mathbf{Y}_2)$
  $\mathcal{L} = \frac{1}{m_1}\sum_{i=1}^{m_1} d(x_i, \hat{x}_i)) + \frac{\omega}{m_2}\sum_{i=1}^{m_2} \max(0, \delta - d(z_i, \hat{z}_i)) \pm \lambda T(\{F_\theta(x_i)\})$ where $\hat{x}_k \in \hat{\mathbf{X}}_1, \hat{z}_\ell \in \hat{\mathbf{X}}_2$
  $\boldsymbol{\Xi}_{j+1} = \boldsymbol{\Xi}_j - \eta_1 \nabla_{\boldsymbol{\Xi}}\mathcal{L}$ or other optim
  $\boldsymbol{\phi}_{j+1} = \boldsymbol{\phi}_j - \eta_1 \nabla_{\boldsymbol{\phi}}\mathcal{L}$ or other optim
  $\mathbf{D} = \nabla_{\boldsymbol{\Theta}} T^\star$
  $\Gamma = \mathbf{D} - \boldsymbol{\Theta}_j(\boldsymbol{\Theta}_j^T\mathbf{D} + \mathbf{D}^T\boldsymbol{\Theta}_j)/2$
  $\boldsymbol{\Theta}'_{j+1} = \boldsymbol{\Theta}_j + \eta_2\Gamma$
  Compute $\mathbf{R}\boldsymbol{\Lambda}\mathbf{S}^T = \text{SVD}(\boldsymbol{\Theta}'_{j+1})$ ▷ where SVD is singular value decomposition
  $\boldsymbol{\Theta}_{j+1} = \mathbf{RS}^T$

Algorithm S2 Algorithm for computing the empirical null distribution

---

Input: Trained encoder F_θ, decoder G_ϕ, reconstruction loss d, and B-DNA test set $\mathbf{X}^B$ consisting of n_B samples.
$\mathbf{Y} = F_\theta(\mathbf{X}^B)$ ▷ Collect the code vectors from B-DNA
Use MCD to robustly estimate $(\hat{\boldsymbol{\mu}}_\mathbf{Y}, \hat{\boldsymbol{\Sigma}}_\mathbf{Y})$ of $\mathbf{Y}$
Compute the MCD based Mahalanobis distance $d_i \in \mathbb{R}_{\geq 0}$ for each sample $\mathbf{y}_i$, $i \in \{1, \ldots, n_B\}$
$\hat{\mathbf{X}}^B = G_\phi(\mathbf{Y})$ ▷ Reconstruct B-DNA
Compute reconstruction loss $\mathbf{r} = d(\mathbf{X}^B, \hat{\mathbf{X}}^B)$. ▷ $\mathbf{r}$ is a vector of residuals with r_i indicating the reconstruction error for sample i.
Use MCD to robustly estimate $(\hat{\boldsymbol{\mu}}_\mathbf{rd}, \hat{\boldsymbol{\Sigma}}_\mathbf{rd})$ on the bivariate collection $\{(r_i, d_i)\}_{i=1}^{n_B}$
Compute the MCD based Mahalanobis distance s_i for each pair (r_i, d_i), $i = 1, \ldots, n_B$ ▷ $\{s_i\}_{i=1}^{n_B}$ forms the null distribution.

Algorithm S3 Algorithm for computing statistic on new observations

---

Input: Trained encoder F_θ, decoder G_ϕ, reconstruction loss d, MCD estimators $(\hat{\boldsymbol{\mu}}_\mathbf{Y}, \hat{\boldsymbol{\Sigma}}_\mathbf{Y})$ and $(\hat{\boldsymbol{\mu}}_\mathbf{rd}, \hat{\boldsymbol{\Sigma}}_\mathbf{rd})$ pre-computed from test set B-DNA, and a Non-B DNA test set $\mathbf{X}^N$ consisting of n_N samples.
$\tilde{\mathbf{Y}} = F_\theta(\mathbf{X}^N)$ ▷ Collect the code vectors of from Non-B DNA
Compute the MCD based Mahalanobis distance $\tilde{d}_i \in \mathbb{R}_{\geq 0}$ for each sample $\tilde{\mathbf{y}}_i$, $i \in \{1, \ldots, n_N\}$ using $(\hat{\boldsymbol{\mu}}_\mathbf{Y}, \hat{\boldsymbol{\Sigma}}_\mathbf{Y})$
$\hat{\mathbf{X}}^N = G_\phi(\tilde{\mathbf{Y}})$ ▷ Reconstruct non-B DNA
Compute reconstruction loss $\tilde{\mathbf{r}} = d(\mathbf{X}^N, \hat{\mathbf{X}}^N)$. ▷ $\tilde{\mathbf{r}}$ is a vector of residuals with $\tilde{r}_i$ indicating the reconstruction error for sample i.
Compute the MCD based Mahalanobis distance $\tilde{s}_i$ for each pair $(\tilde{r}_i, \tilde{d}_i)$, $i = 1, \ldots, n_B$ using $(\hat{\boldsymbol{\mu}}_\mathbf{rd}, \hat{\boldsymbol{\Sigma}}_\mathbf{rd})$

A.3 Architecture & Training Details

The `GoFAE-DND` architecture consists of several linear and 1D convolution layers with ELU activation. The data was structured to have 2 channels (forward, reverse) each consisting of a sequence of 50 base pairs. The Adam optimizer was used for both the first stage of encoding and the decoder.

The learning rate was set at $1e-3$ with $\beta_0 = .9$ and $\beta_1 = .999$. Riemannian stochastic gradient descent was used for updating the the final linear layer of the encoder (denoted Stiefel in Table S1) with an initial learning rate of $1e-3$. Both encoder and decoder used learning rate schedules which decreased after no improvement on the B-DNA validation reconstruction loss. The Riemannian SGD used the 1 cycle learning rate scheduler with a maximum learning rate of $5e-3$. The models were trained with minibatch size of 128 for 25 epochs using PyTorch on a high performance cluster. The parameter $\omega = 0.5$ was fixed for all experiments.

Cross-validation using grid-search was conducted for hyper-parameter selection. On the simulated data the contrastive weight $\delta \in \{20, 25, 30, 35\}$, $n_z \in \{32, 64\}$, and $n_h = 32$. The test statistic regularization coefficient λ was fixed at 0.5. The model had a relatively easy time with the transformation. For the experimental data, $\delta \in \{30, 35, 40, 45\}$, $n_z \in \{32, 64\}$, and $\lambda = 35$, and $n_h = 32$.

Table S1. GoFAE-DND architecture

Dataset	Optimizer		Architecture
Nanopore	Adam	Input	2
		Encoder	Conv1d(2, n_h, kernel_size=(5,), stride=(1,), padding=(2,))<br>ELU(alpha=1.0, inplace=True)<br>Conv1d(n_h, n_h, kernel_size=(5,), stride=(2,), padding=(1,))<br>ELU(alpha=1.0, inplace=True)<br>Flatten(start_dim=1, end_dim=-1)
	RSGD	Input	$n_h \times 24$
		Stiefel	32 Orthogonal initialization<br>Linear(in_features=$n_h \times 24$, out_features=n_z, bias=False)
	Adam	Input	n_z
		Decoder	Linear(in_features=n_z, out_features=$n_h \times 24$, bias=True)<br>ELU(alpha=1.0, inplace=True)<br>ConvTranspose1d(n_h, n_h, kernel_size=(5,), stride=(2,), padding=(1,))<br>ELU(alpha=1.0, inplace=True)<br>ConvTranspose1d(n_h, n_h, kernel_size=(3,), stride=(1,), padding=(1,))<br>ELU(alpha=1.0, inplace=True)<br>ConvTranspose1d(n_h, 2, kernel_size=(4,), stride=(1,), padding=(1,))

A.4 Description of Comparison Methods

Isolation forest (IF) or iForest (Liu *et al.*, 2008) is an unsupervised anomaly detection method that leverage data sub-samples and an ensemble of isolation Trees to detect anomalies. The general approach makes an assumption that there are few anomalies in the data that are likely to have attributes that distinguish them from other data samples. The method repeatedly selects a feature at random and a split point and then builds a binary tree on a selected subset of data. The assumed qualities of anomalies (i.e. being few and distinct) make them susceptible to isolation in these trees such that they end up closer to the root compared to the non-anomalous points. For the evaluation of each data sample, an anomaly score is computed as the average path length from the root to the data sample. This average path length is thresholded to compute anomalies.

Local outlier factor (LOF) (Breunig *et al.*, 2000) is an unsupervised novelty detection method suitable for multidimensional datasets. LOF is related to density-based clustering, where the goal is computing a local outlierness score (i.e. LOF) for each sample based on its k nearest neighbors. A sample's $k-$distance is the distance between that sample and its farthest neighbor ($k-$th neighbor). Then the reachability distance is calculated between two samples as the maximum of their $k-$distance and their actual distance. The intuition is that if two samples are close enough, their reachability distance will be equal to their $k-$distance, however, if they are far away, this value will be equal to their distance. Then the local reachability density of the sample will be equal to the inverse of the average reachability distance of the sample and all data points in its neighborhood. Finally, to call anomalies, LOF evaluates how different a sample's local reachability density is with respect to its neighbors.

One-class support vector machines (one-class SVM) (Schölkopf *et al.*, 2001) is an unsupervised anomaly detection method that extends the support vector algorithm for unlabeled data. Like previous methods, one-class SVMs aim to group non-anomalous data and then call anomalies based on those data samples that do not fall within the non-anomalous class. Unlike the previous methods, one-class SVMs compute a minimally-sized hypersphere containing the non-anomalous data; data samples that do not fall within the hypersphere are considered anomalies. By leveraging the kernel trick, one-class SVMs are among the more flexible anomaly detection methods.

A.5 Interval Wise Testing (IWT)

IWT is a hypothesis testing procedure often used for functional data analysis of -omics data Cremona (2018). It performs a non-parametric permutation test on all possible combinations of curves and scales with the goal of detecting significant differences between two sets of regions. The input to IWT are real-valued measurements in predefined intervals and a resolution (we use single basepair resolution in this work). The general IWT procedure is:

1. Estimate the empirical distribution of the test statistic under null, conditioned on the data for all possible permutations of the observed curves on any sub-interval in the region of interest and control region.
2. Compute the (two-sided) test p-value, which is the proportion of permutations that lead to a test statistic greater than or equal to the one evaluated on the original data.
3. Adjust the p-value curve.
4. Identify locations with a significant difference in motif-containing versus motif-free windows.

B Supplementary Results

B.1 FAST5 file format

The FAST5 file format is a schema based on the hierarchical data format 5 (HDF5) and defined by ONT to store raw current-signal data generated by ONT devices. It contains groups and members similar to directories and files in a file system. Any of the groups or members (HDF5 objects) can optionally contain attributes, which are represented as key-value pairs. A single FAST5 file can contain information about one or more reads. Essential sequencing information is stored per read, including several quantities important for this work:

- Digitization: the number of quantization levels in the Analog to Digital Converter.
- Offset: The ADC offset error: this value is added when converting the signal to pA.
- Range: The full scale measurement range in pA.
- Sampling rate: The number of data points collected per second (Hertz).
- Block Stride: The number of signal samples allocated for each stride.
- Move table: The number of base shifts in each stride block (usually either 0 or 1).

The block stride and move table are written into the file by the basecalling procedure.

B.2 Basecalling using Albacore

We used version 2.3.1 of Albacore to perform basecalling.

```
read_fast5_basecaller.py -f FLO-PRO002 -k SQK-LSK109 --input $source --save_path $destination --
    output_format fastq,fast5 -t 48 --recursive --config r941_450bps_linear_prom.cfg;
```

B.3 Tombo Re-squiggle

We used version 1.5.1 of Tombo to perform re-squiggling.

```
tombo resquiggle $source/workspace/pass/ hg38.fa --dna --overwrite --basecall-group Basecall_1D_001
    --include-event-stdev --failed-reads-filename tombo_failed_reads.txt --processes 48;
```

B.4 Filtering

We filtered reads based on SAM flags and mapping quality; we only consider the reads that have scores higher than or equal to 20.

```
# for forward strand:
samtools view merged_chr1+.bam chr1:start-end -F 0xF14 -o $destination/chr1+.bam;
bedtools bamtobed -i $destination/chr1+.bam > $destination/chr1+.bed;

# for reverse strand:
samtools view merged_chr1-.bam chr1:start-end -F 0xF04 -f 0x10 -o $destination/chr1-.bam;
bedtools bamtobed -i $destination/chr1-.bam > $destination/chr1-.bed;
```

B.5 Additional data processing details

B-DNA window construction. After B-DNA windows were extracted from genomic intervals without non-B DNA motifs, we computed 1,200bp regions without a non-B DNA motif and trimmed the flanking 100bp regions bordering non-B motifs to reduce the possibility that non-B DNA structures could affect B-DNA TTs. Additionally, we removed any reads with starting or terminal positions within B-DNA regions. We divided these regions into non-overlapping windows of 100bp and extracted the median translocation times for each strand and position to form the B-DNA dataset $\mathcal{X}^B$.

Subsampling B-DNA. Let the size of non-B DNA and B-DNA in the concatenated training and validation set be $|X^N tr, val|$ and $|X^B tr, val|$ respectively. Then, we randomly sampled min $\left(|X^B tr, val|, 5 \times |X^N tr, val|\right)$ data points from the B-DNA data to form the train and validation sets.

Simulation details. The functions to generate G-quadruples were $-((a \cdot t)^2 + b)$ and $(a \cdot t)^2 + b$ for forward and reverse strands, where $a = 0.05$. For short tandem repeats, we used sinusoidal patterns of $b \sin(c \cdot t)$ where $c = 0.3$. In both datasets $b \in \{0.5, 0.75, 1\}$ and $t \in (-25, 25)$ and the motifs were 5bp, 10bp or 20bp long. After generating samples from these functional forms, we added Gaussian noise $\mathcal{N}(0, 1)$ to each sample. The B-DNA data was sampled from Gaussian noise $\mathcal{N}(0, 1)$. We simulated 200,000 B-DNA windows and 20,000 non-B DNA windows in each dataset and varied the amount of true non-B DNA structures in the non-B labeled samples $\in$ {0.05,0.1,0.25}.

Let the size of the target non-B DNA and B-DNA simulated datasets and the non-B ratio be $|X^N|$, $|X^B|$ and r respectively. To simulate the experimental setting, we keep only $|X^N| - M$ samples from non-B data, where $M = |X^N| - r \times |X^B|$. Then, we sample M new B-DNA windows from the B-DNA simulator, relabel them as our desired non-B DNA type and mix them with the non-B DNA data, while keeping the true labels of samples for evaluation. (e.g. if $|X^N| = 20,000$, $|X^B| = 200,000$, and $r = 0.05$, we keep 1,000 actual non-B DNA samples in the data and sample 19,000 new B-DNA windows).

B.6 Additional figures and tables

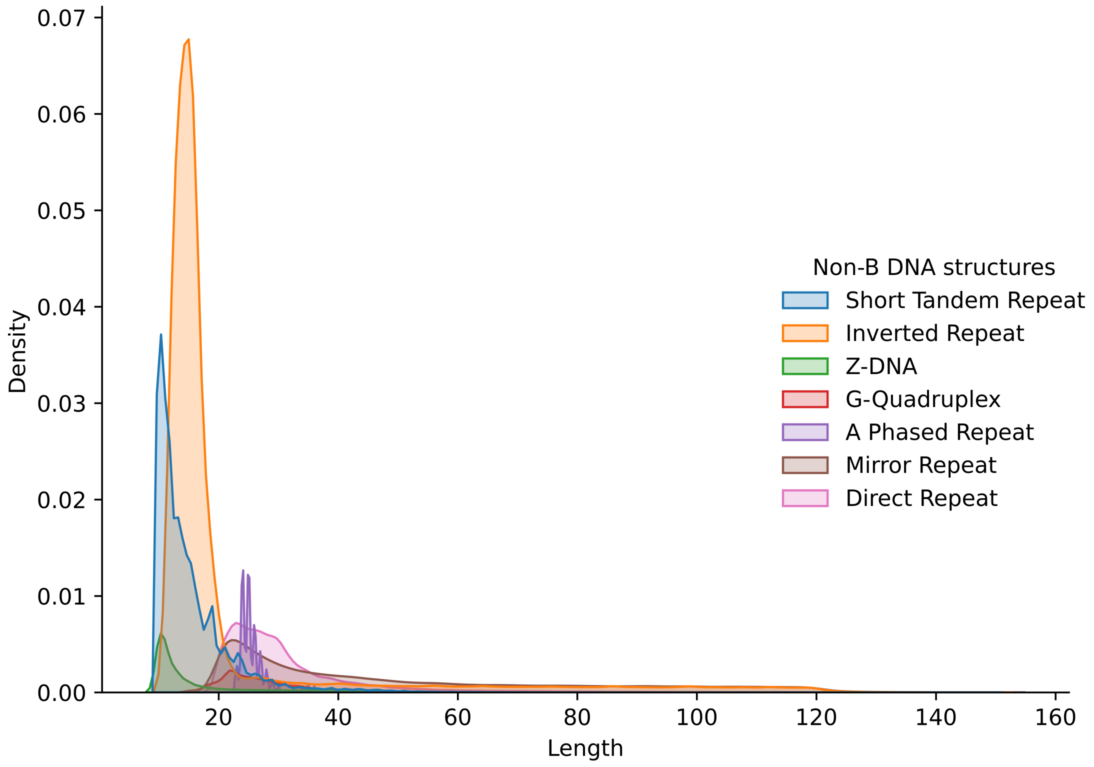

Fig. S1: **Density plot of the length of the motifs across non-B DNA types in DB prior to preprocessing.** We cut out the motifs with a length that is more than 150bp. The removed motifs account for 0.12% of the total.

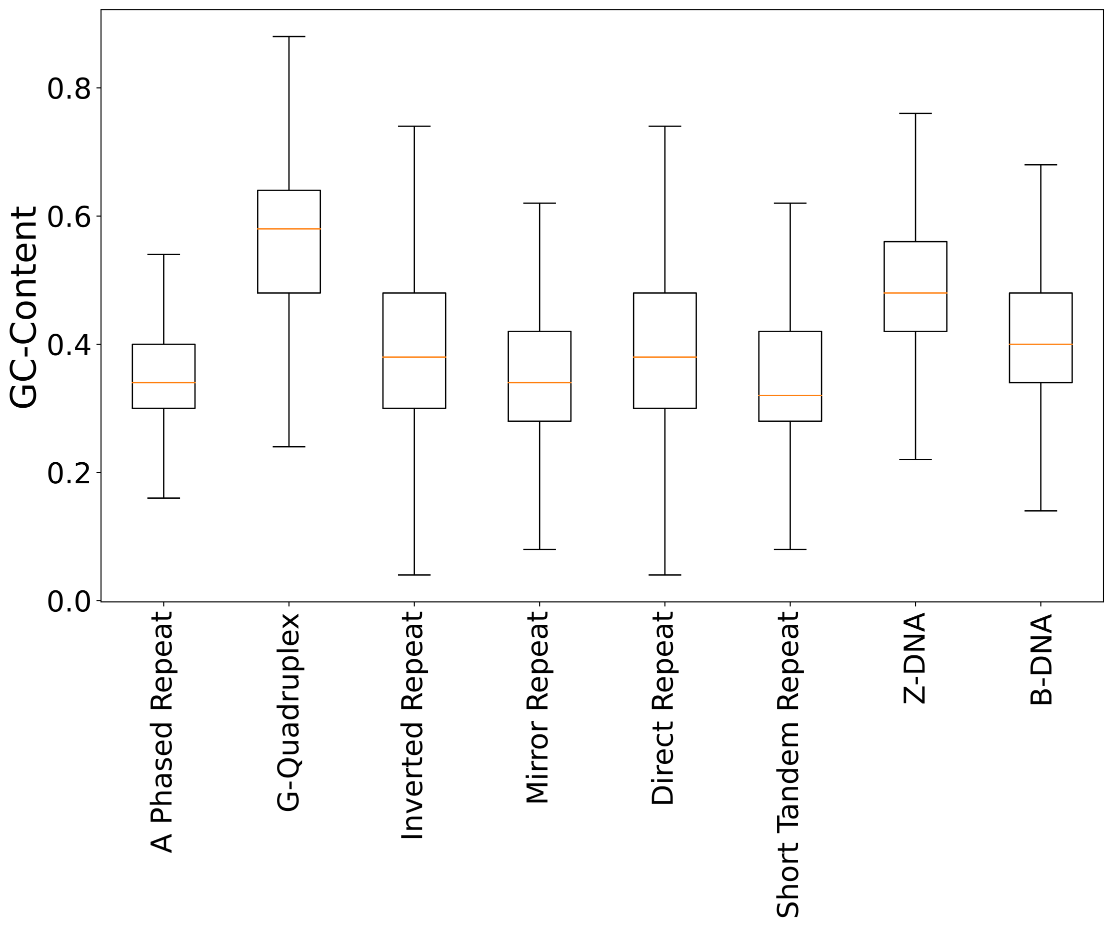

Fig. S2: **GC-Content of datasets.** This figure shows the distribution of GC-content in non-B and B-DNA windows in the experimental data.

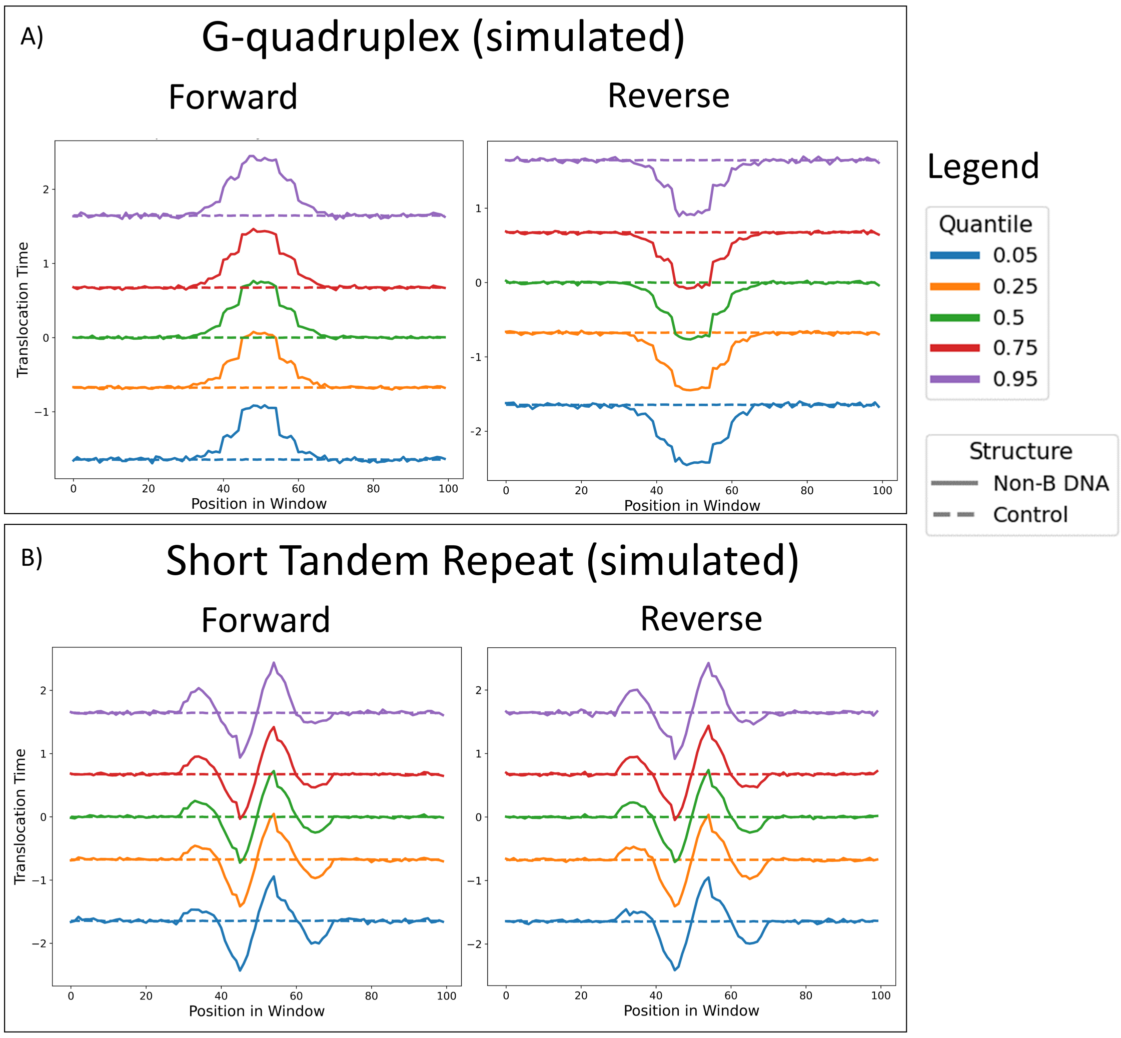

Fig. S3: **Comparison between B and non-B DNA translocation time in simulated windows.** The 0.05, 0.25, 0.5, 0.75, 0.95 quantiles of translocation times for (solid line) (A) G-quadruplexes, (B) short tandem repeats show deviation from B-DNA control windows (dashed line). The x axis gives the position relative to the window start and the y axis is the translocation time.

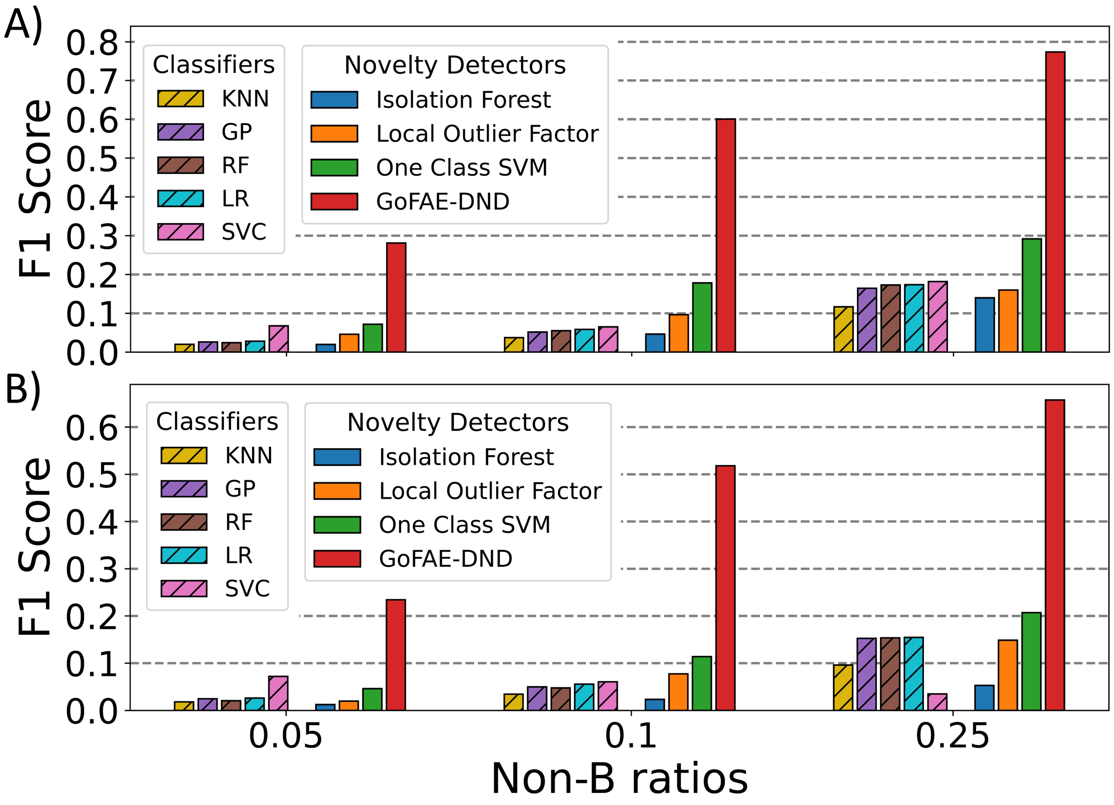

Fig. S4: **A comparison of F_1 scores for classifiers and non-B novelty detection methods in synthetic data for (A) G4 and (B) short tandem repeats.**

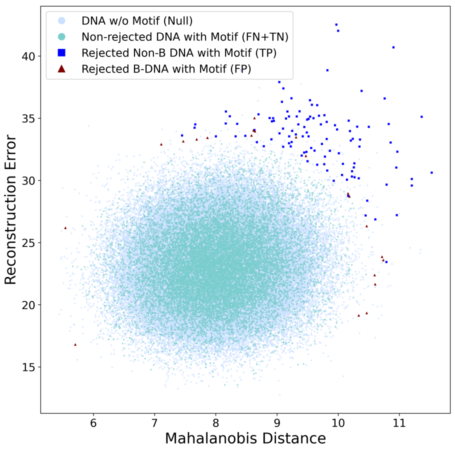

a) Short Tandem Repeat at 5%

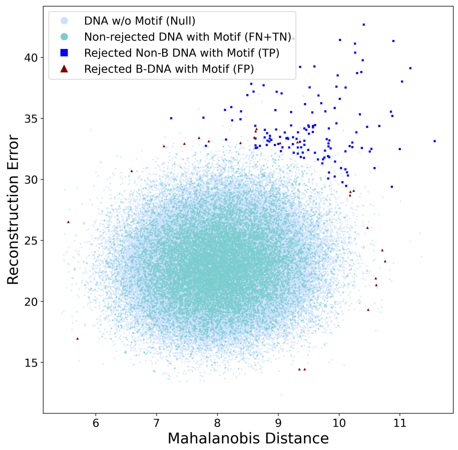

b) G-Quadruplex at 5%

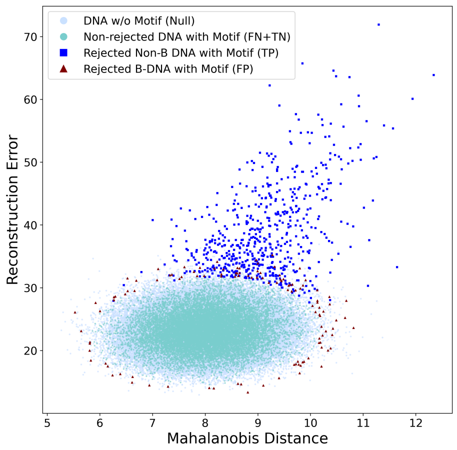

c) Short Tandem Repeat at 10%

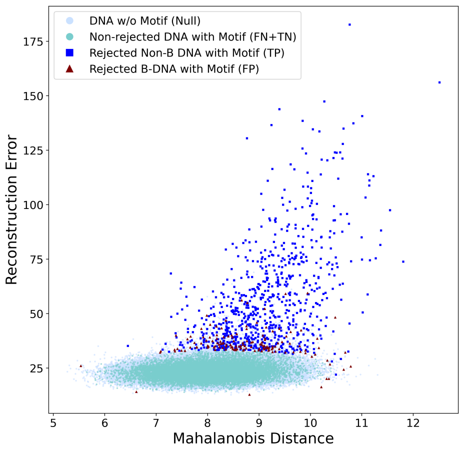

d) G-Quadruplex at 10%

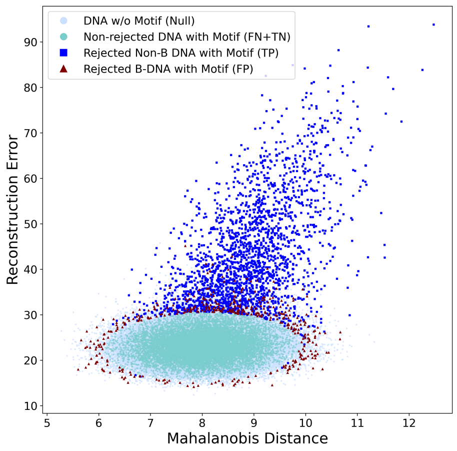

e) Short Tandem Repeat at 25%

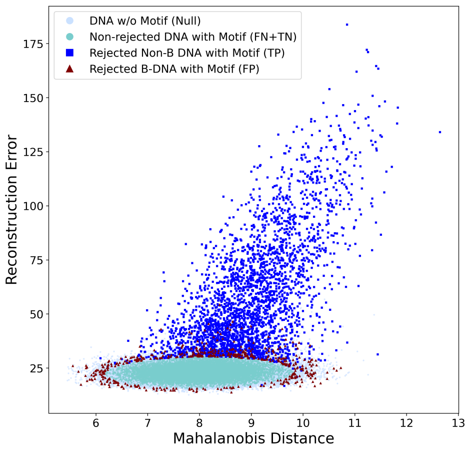

f) G-Quadruplex at 25%

Fig. S5: Figs.(a-f) are scatter plots of simulated DNA with and without a motif at 3 different ratios. The test sets, all (●,●,■,▲), are passed through the network to produce a code vector and reconstruction error. DNA without a motif serves as the null distribution and is marked by (●). DNA considered to have a motif includes (●, ■, ▲). Using the simulated ground truth labels and controlling FDR at level $\alpha = .2$, (●) denotes observations not rejected, (■) denotes observations which are correctly rejected (true positives (TP)), and (▲) denotes observations incorrectly rejected and deemed novel (false positives (FP)). Note how the FP points are more evenly distributed in the tails of the null distribution, acting more like outliers with no systematic deviation versus the TP points which present in a different manner–generally clustering together. However, we stress that this does *not* imply the TP points are all deviating for the same reason.

Method	Parameters
Isolation Forests	max sample $\in \{256, 500, 100, 2000, 3000\}$
SVM One Class	kernel $\in$ {linear, poly, rbf, sigmoid }
Local Outlier Factor	Number of neighbors $\in \{10, 20, 40, 100, 200\}$

Table S2. Hyperparameter settings for each method on experimental.

Dataset	Median GC-Content	Mean GC-Content
A Phased Repeat	0.340%	0.352%
G-Quadruplex	0.580%	0.556%
Inverted Repeat	0.380%	0.387%
Mirror Repeat	0.340%	0.357%
Direct Repeat	0.380%	0.392%
Short Tandem Repeat	0.320%	0.350%
Z-DNA	0.480%	0.492%
B-DNA	0.400%	0.414%

Table S3. GC-content of datasets. This table shows the mean and median GC-content across different non-B types and B-DNA datasets.

**non-B DNA**	**count**	% **non-B**
A phased repeat	75,995	0.049
G-quadruplex	40,630	0.026
Inverted repeat	987,383	0.636
Mirror repeat	82,882	0.054
Direct repeat	52,461	0.034
Short tandem repeat	296,348	0.191
Z-DNA	17,019	0.011

Table S4. The number of non-B DNA windows in the experimental data.

Datasets	Isolation Forest	Local Outlier Factor	One Class SVM	GoFAE-DND
A Phased Repeat	0 (0.00%)	0 (0.00%)	0 (0.00%)	5,137 (8.45%)
G-Quadruplex	3,003 (9.24%)	3 (0.00%)	12,364 (38.04%)	11,334 (34.87%)
Inverted Repeat	3 (0.00%)	0 (0.00%)	33,669 (4.26%)	41,950 (5.31%)
Mirror Repeat	0 (0.00%)	0 (0.00%)	0 (0.00%)	7 (0.01%)
Direct Repeat	0 (0.00%)	0 (0.00%)	0 (0.00%)	66 (0.16%)
Short Tandem Repeat	1 (0.00%)	143 (0.06%)	44,212 (18.65%)	112,631 (47.51%)
Z-DNA	0 (0.00%)	0 (0.00%)	0 (0.00%)	253 (1.86%)

Table S5. Novelties discovered at $\alpha = 0.2$ FDR control. The counts of non-B DNA windows in the test set are 60,797, 32,504, 789,907, 66,306, 41,969, 237,080, 13,617 for A Phased repeat, G-quadruplex, Inverted, Mirror, Direct, and Short Tandem repeats and Z-DNA, respectively. The percentages of discoveries are in parentheses.

		Classifiers					Novelty Detectors			
		KNN	GP	RF	LR	SVC	IF	LOF	SVM	GoFAE-DND
G-Quadruplex	0.05	0.0199	0.0261	0.0244	0.0279	0.0676	0.0198	0.0461	0.0718	**0.2812**
	0.1	0.0371	0.0516	0.0549	0.0584	0.0648	0.0462	0.0966	0.1783	**0.6008**
	0.25	0.1166	0.1645	0.1728	0.1736	0.1818	0.1399	0.1602	0.2920	**0.7737**
Short Tandem Repeat	0.05	0.0182	0.0247	0.0207	0.0262	0.0722	0.0124	0.0198	0.04612	**0.2344**
	0.1	0.0345	0.0499	0.0478	0.0556	0.0607	0.0235	0.0776	0.1140	**0.5181**
	0.25	0.0962	0.1525	0.1537	0.1546	0.0349	0.0528	0.1485	0.2070	**0.6573**

Table S6. A comparison of F_1 scores in simulated data for GoFAE-DND and competing classifiers and novelty detection methods.
